# Insights in altered eating behaviors in women with Ehlers-Danlos syndromes

**DOI:** 10.1007/s40519-026-01872-2

**Published:** 2026-05-21

**Authors:** Carolina Baeza-Velasco, Elisabet Tasa-Vinyals, Paola Espinoza, Sébastien Guillaume, Maria Soledad Mora, Antonio Bulbena, Sonia Lorente

**Affiliations:** 1 Laboratoire de Psychopathologie et Processus de Santé, Université Paris Cité, F-92100 Boulogne Billancourt, France; 2 Department of Emergency Psychiatry and Acute Care, CHU Montpellier, Montpellier, France; 3 Institute of Functional Genomics, University of Montpellier, CNRS, INSERM, Montpellier, France; 4 Eating Disorders Section, Department of Child and Adolescent Psychiatry and Psychology, Institut Clinic de Neurociències, Hospital Clínic de Barcelona, Barcelona, Spain; 5 Fundació de Recerca Clínic Barcelona-Institut d’Investigacions Biomèdiques August Pi i Sunyer (IDIBAPS), 08036 Barcelona, Spain; 6 Faculty of Medicine, University of Barcelona, 08036 Barcelona, Spain; 7 Universitary Hospital Sagrado Corazón, Quirón Salut Group, Barcelona, Spain; 8 Department of Clinical and Health Psychology, Universitat Autònoma de Barcelona, Barcelona, Spain; 9 Departament of Psychiatry and Forensic Medicine, Universitat Autònoma de Barcelona, Barcelona, Spain; 10 Department of Psychobiology and Methodology in Health Sciences, Universitat Autònoma of Barcelona, Edifici BBellaterra (Cerdanyola del Vallès) Espanya, 08290 Barcelona, Spain

**Keywords:** Ehlers-Danlos syndrome, Joint hypermobility, Disordered eating, Gastrointestinal problems, Somatosensory amplification, Food allergies, Fear, BMI

## Abstract

**Background:**

Recent research on Ehlers-Danlos syndromes (EDS) highlights a high frequency of disordered eating behaviors and Body Mass Index (BMI), especially in the hypermobile subtype. These problems might be secondary to EDS symptoms and comorbidities such as gastrointestinal (GI) problems, food allergies and oral pain, but also to psychological factors. To test this hypothesis, we explored the above-mentioned somatic and psychological features and their connexions with eating behavioural outcomes and BMI.

**Method:**

Women with self-reported EDS (n = 121) completed an online survey assessing GI problems, food allergies, oral pain, somatosensory amplification, painful and fearful eating, eating avoidance and restriction, body satisfaction, and BMI. Path Analysis model was performed to examine the relationships among the variables.

**Results:**

Significant relationships between GI problems and food allergies (B = 2.36, p = 0.035), fearful eating (B = 0.10, p = 0.043), painful eating (B = 0.02, p = 0.001) and somatosensory amplification (B = 1.01, p < 0.001) were observed. Also, positive relationships between painful and fearful eating (B = 0.76, p < 0.001), and fearful and avoidant eating (B = 0.78, p < 0.001).

**Conclusions:**

These results support the idea that certain somatic symptoms common in EDS would lead to fear and avoidant responses to food. Further research is needed to better understand the link and directionality between abnormal connective tissue and disordered eating behaviors.

**Level of evidence IV:**

Evidence obtained from observational-descriptive studies, such as case series, individual case reports, and descriptive cross-sectional studies.

## Introduction

The Ehlers-Danlos syndromes (EDS) are a group of inherited connective tissue disorders characterized by abnormal collagen synthesis affecting all supporting structures of the body. Fourteen EDS subtypes are currently described [1, 2] however the hypermobile EDS subtype (hEDS) is diagnosed in most cases (80–90%) [3]. In addition to being the most frequent EDS, hEDS is the only one for which a genetic abnormality has not yet been identified. It is hypothesized that hEDS is associated with small effect polygenic risk factors and environmental exigencies [4]. Thus, hEDS diagnostic is based on clinical criteria, while in the case of the other subtypes, genetic testing can confirm the diagnosis [3].

All EDS subtypes share features such as joint hypermobility (JH), skin hyperextensibility, and tissue fragility [2, 5]. Though, given the wide distribution of collagen in body tissues, people with EDS may have problems in different organs and body systems in addition to problems in the body’s supporting structures [6]. In this sense, among the extra-articular problems, gastrointestinal (GI) disorders are very common, especially in people with hEDS [7–11]. These include altered bowel function, dyspepsia, gastro-esophageal reflux, dysphagia, recurrent abdominal pain, and constipation/diarrhea [10]. Food allergies or intolerances have also been reported as higher in people with hEDS than in controls [8].

Although the pathophysiology of GI disorders in hEDS remains unclear, some plausibly aspects involved include the degree of laxity of connective tissue, dysautonomia, treatment (medication) itself and psychological variables [12]. In this sense, a tendency to experience an anodyne or mild somatic sensation as intense, noxious, and disturbing (somatosensory amplification), has been frequently reported in people with symptomatic JH [13] and can exacerbate GI symptoms.

On the other hand, the association between functional GI problems and eating disorders (ED) is well-known [14]. In this regard, some case reports suggest that undiagnosed, untreated or persistent EDS symptoms might lead to a secondary ED diagnosis, including severe disorders such as anorexia nervosa or bulimia nervosa [15–17]. At the same time, there is also case-report evidence of patients receiving misdiagnoses of EDs that are later ruled out after confirmation of hEDS with significant GI symptoms explaining all of the symptomatology linked to the ED criteria [18]. Systematic studies exploring ED in people with hEDS are extremely rare. Recently, we reported a significantly higher prevalence of self-reported ED history and a higher risk of current ED in people with EDS compared to controls [8]. In the same vein, another study [19] reported that 37.9% (n = 680) of patients with hEDS or hypermobility spectrum disorders (an umbrella category describing symptomatic JH which do not fulfill criteria for hEDS) screened positive for avoidant/restrictive food intake disorder (ARFID), while the prevalence of clinically diagnosed ARFID in the general population is estimated between 0.3 and 15.5% [20].

In our view and clinical experience, with its frequent comorbid GI problems and other symptoms globally affecting the act of eating, hEDS represents a potential fertile ground for the development of altered eating behaviors and even ED [15]. Moreover, oral mucosa fragility and temporomandibular disorders probably due to JH have also been described in hEDS [21, 22]. These alterations may affect chewing and lead to oral pain. In addition, the above-mentioned food allergies/intolerances as well as GI symptoms such as dysphagia and abdominal pain arguably make the eating experience less safe, less pleasant and less satisfactory. As we stated in a previous work [15], it is possible to extrapolate the fear of pain and movement frequently observed in people with chronic musculoskeletal pain leading to avoid physical activity, to the fear of eating due to oral and visceral pain. This can be the case of a subgroup of hEDS patients who may develop avoidance-altered eating behaviors as a response to pain or discomfort. In addition, it must be noted that hEDS, like other chronic pain conditions [23], can be linked to a negative body image, especially considering that hEDS is associated with body awareness particularities (e.g. impaired proprioception) and external signs of disease (e.g. splinting, wheelchair).

Speculatively, all these physical and psychological aspects might help explain the increased rate of eating difficulties, abnormal BMI and prevalence and clinical presentation of EDs in hEDS. However, to date we are not aware of any study having comprehensively and integratively explored the association of these specific variables on negative eating-related outcomes.

Therefore, the aim of the present work was twofold and should be considered exploratory and hypothesis-generating in nature. Firstly, to describe the relationship between GI problems, food allergies, oral pain and somatosensory amplification, and secondly, to describe the link between these variables and painful, fearful and avoidant eating, exploring in turn how all these relationships may affect corporality by using BMI as anthropometric variable.

## Methods

### Design, setting and participants

This is an observational cross-sectional study. Inclusion criteria were being adult female (aged 18 years or older), and self-reported diagnosis of EDS. Exclusion criteria were related to language barriers. The participant sample consisted of a sub-sample of a previous study [8] recruited through the Spanish National EDS Patient Association (*Asociación Nacional del Síndrome de Ehlers-Danlos e Hiperlaxitud,* ANSEDH). Though the association does not require medical proof of diagnosis to issue member status, it is a non-profit organization specifically aimed at EDS patients. Members do actively request their admission on the mere basis of their diagnosis, usually shortly after receiving it. Therefore, it is unlikely for any person who has not received a diagnosis of EDS from their physician to become aware of the association and eventually be granted membership. Participation, which consisted of the completion of a self-administered online survey, was voluntary and anonymous. Participants did not receive any economic, healthcare-related or other kind of compensation in exchange for their time and/or data and were duly informed of the possibility of withdrawing their consent at any time if they wished to do so. For all variables, a short description was included in the form to clarify key concepts and ensure consistent data collection from participants. Participants were also provided with contact e-mails from the research team in case they needed further clarification.

### Variables and instruments

Sociodemographic characteristics (age, country of residence, marital status, number of children, educational and work status) and clinical characteristics (EDS subtype; age at symptom onset (first or prodromic symptoms often being joint hypermobility, chronic pain, or joint instability) regardless of whether a formal diagnosis had been established at that time; formal diagnosis; food allergies and/or intolerances, and personal/family history of any relevant health conditions, particularly ED) were collected. Body Mass Index (BMI) was also recorded, calculated on the basis of the self-reported data on height and weight, and then categorized according to the World Health Organization criteria [24].

The Self-reported Screening Questionnaire for Joint Hypermobility Syndrome (SQCH) [25] was used to confirm symptomatic JH and to exclude non-eligible participants. The questionnaire is composed of seven items, five of which correspond to the 5-point questionnaire for JH [26] plus two extra-articular features (abnormal scarring and easy bruising). A cutoff score of ≥ 3 indicates the presence of JH syndrome. This instrument shows adequate metric properties, including a scale of reliability, as measured by Cronbach’s α coefficient α = 0.84 [25]. The Cronbach α coefficient in this study was α = 0.40.

The SCOFF [27, 28] in its Spanish version [29] was used for ED screening. The acronym SCOFF relates to one of the words in each of the five true–false items composing the English version of the questionnaire, i.e., Sick, Control, One, Fat, Food. The instrument can be administered in less than 1 min, and the items address the core features of ED such as recent and significant weight loss, distorted body image, or whether the person feels that food controls their life. Scoring consists of adding up the number of affirmative (“true”) answers. Respondents scoring ≥ 2 are considered at risk of an ED. The SCOFF total score is also used for comparing the severity of eating problems between different populations [30, 31]. The Spanish version has been successfully validated in Spain in a study that corroborated the psychometric properties of the aforementioned cutoff point, which presented 97.7% sensitivity and 94.4% specificity for detecting ED in primary care settings with adult female patients [29]. The Cronbach α coefficient obtained in this study was moderate but acceptable α = 0.57.

GI problems were assessed by means of the Patient Assessment of Upper Gastrointestinal Disorders-Symptom Severity Index (PAGI-SYM) [32] in its Spanish version [33]. The PAGI-SYM is a 20-item instrument consisting of 6 subscales: heartburn/regurgitation, postprandial fullness/early satiety, nausea/vomiting, bloating, upper abdominal pain and lower abdominal pain. Items are presented as short sentences that succinctly describe core symptoms in everyday life (e.g., feeling unpleasantly fed up, regurgitating, being unable to finish a whole meal). Each item is presented as a 6-point Likert scale. Higher scores imply more severe gastrointestinal problems. The Spanish version has shown good reliability (Cronbach’s α = 0.87) and convergent validity, along with a 6-factor structure analogue to the one described by the original authors [33]. The Cronbach α coefficient in the present study was α = 0.91 for Heartburn subscale; α = 0.80 for Postprandial fullness; α = 0.85 for Nausea/vomiting; α = 0.91 for Bloating and Upper abdominal pain and α = 0.95 for Lower abdominal pain.

Somatosensory amplification was measured by means of the Somatosensory Amplification Scale (SSAS) [34] in its Spanish version [35]. This scale consists of 10 items describing everyday-life situations where somatosensorial amplification might play a visible role, including both internal and external stimuli, some of them related to the eating experience (e.g., *I am often aware of various things happening within my body; I am quick to sense the hunger contractions in my stomach*; *I have a low tolerance for pain*). Answers to each item are graded on a 5-point Likert scale. The original authors demonstrated that SSAS scores were normally distributed, had acceptable test–retest reliability and internal consistency, and were related to hypochondriasis [34]. Moreover, the Spanish adaptors significantly correlated SSAS scores with relevant factors such as bodily preoccupations, health habits and disease phobia [35]. The Cronbach α coefficient in this study was α = 0.71.

The Three-Factor Eating Questionnaire (TFEQ) [36] was administered in its Spanish version [37] to assess restricted eating. This instrument consists of 17 items graded on a 4-point Likert scale, a higher score reflecting a greater degree of pathology, plus an extra 18th item graded on an 8-point Likert scale from no restriction at all to totally restricted eating. In the Spanish validation, the factor analysis confirmed a three-factor structure: restricted eating, uncontrolled eating, and emotional eating. Items inquire about manifestations of restraint (e.g., avoiding eating or storing certain food types at home in fear of becoming fat), uncontrolled eating (e.g., inability to stop eating once started, appetite highly increased by the vision of others eating) and emotional eating (e.g., eating in situations of fear or loneliness regardless of physiological sensations of hunger, or mistaking such sensations with negative emotions). In the Spanish validation study, the instrument showed a good internal consistency with Cronbach’s α coefficient values ranging between 0.75 and 0.87 [37]. The Cronbach α coefficient in this study was α = 0.85 for restricted eating.

Oral pain was assessed with the physical pain dimension of the Oral Health Impact Profile (OHIP-49) [38] in its Spanish version [39]. The OHIP-49 is a well-known instrument consisting of 49 items in total of which 9 (those numbered 10 to 18 in the original scale) correspond to the physical pain dimension. Items are presented in a 5-point Likert-like format. Greater scores shall be interpreted as more severe and disabling symptomatology. López and Baelum, authors of the Spanish version [39], found fair-to-good internal consistency values for the OHIP-49 (Cronbach’s α = 0.90) and its 7 domains, including physical pain (Cronbach’s α = 0.67), as well as suitable convergent and discriminative validity. The Cronbach α coefficient in this study was α = 0.89.

Painful eating, fearful eating and avoidant eating were assessed through direct 5-point Likert-like scales directly asking participants to what extent they experienced pain during eating, were afraid of eating because of pain or discomfort, and actively engaged in eating avoidance related to pain or discomfort. Higher scores imply greater symptom severity.

Body satisfaction was measured using three 10-point Likert-like items based on the Spanish adaptation of the Body Image Questionnaire [40] and later adapted and validated [41]. Its three items assess the different dimensions of the construct of body image [42]. Specifically, participants were asked to what extent they felt (a) satisfied with and (b) worried by their body image, and (c) to what extent they made an active effort to change their bodies or body parts they were not happy with. Item (a) was thus a direct item while (b) and (c) were reverse: that being so, answers given by subjects to (b) and (c) were recalculated as inverse scores (10 minus [numeric provided answer in the Likert-like scale]), while answers given to (a) were directly used. Answers to the three items were eventually combined in a simple mean, which was treated as a generic score for body satisfaction. The Cronbach α coefficient in this study was α = 0.66.

### Ethical considerations

This study was conducted according to the principles expressed in the Declaration of Helsinki of 1975, revised in 1983, and in full consideration of the Organic Law 15/1999 of December 13 on protection of personal data. Ethical approval was obtained from the *Ethics Committee on Animal and Human Research* of the Universitat Autònoma de Barcelona in September 2019 (registration number ID CEEAH 4799). All participants provided written consent before beginning the online survey.

### Statistical analysis

The statistical analyses were carried out with STATA 16.1 (StataCorp LLC) and reported according to the recommendations of the “Statistical Analyses and Methods in the Published Literature” (SAMPL guidelines) [43].

Data normality distribution was tested by Shapiro Wilk test, skewness, and kurtosis. Test normality for the outcome variable (i.e., BMI) was z = 5.393, p < 0.001, skewness was 1.3 and kurtosis was 6.9, absolute values, suggesting a moderate deviation of normality [44]. A descriptive analysis of the sociodemographic and health variables was performed and overlaps among predictors for the structural model were examined with bivariate Spearman’s correlations, and the variance inflation factor (VIF) for collinearity.

A Path Analysis model was performed to examine the relationship among the predictors of gastrointestinal problems, food allergies, somatosensory amplification, oral pain, painful eating, fearful eating, avoidant eating, body satisfaction and restricted eating, and the outcome variable BMI. The method of estimation was the Maximum Likelihood (ML), which is robust to moderate non-normality distribution for continuous outcome variables as defined by Weston and Gore. Model goodness-of-fit was assessed using the Likelihood Ratio Chi Squared (Chi2), the Standardized Root Mean Square Residual (SRMR), the Root Mean Squared Error of Approximation (RMSEA), the Comparative Fit Index (CFI), and the Tucker-Lewis index (TLI). Smaller values of SRMR and RMSEA suggest a good model fit, whereas larger values of CFI and the TLI larger values indicate better model fit [45]. Modification Indices (MIs) were applied to help evaluate and select specific paths for the best-fitting model.

## Results

A total of 265 participants completed the questionnaire. Of these, 121 were women who self-reported a diagnosis of EDS and were included in the present study. Table 1 shows the socio-demographic and medical history variables of participants. The mean age was m = 40.2, SD (10.7). Regarding marital status, 62.8% reported living with a partner. Regarding education and employment status, 48.8% of the participants were graduates and 15.7% were postgraduates, but only 13.5% were employed. Regarding clinical data, more than half of the EDS patients, 60.3%, reported having been diagnosed with hypermobile EDS, 9.9% with classic EDS, 1.7% with vascular EDS, 9.1% with other forms, and 19% reported not knowing their subtype. A normal BMI was found in 62% of the participants. Other features of medical history were the allergies, reported by the 51.2% of the participants, and other pathologies, such as fibromyalgia, 18.2%, obesity, 21.5%, anorexia, 15.7%, and bulimia, 12.4%.

**Table 1:** Sociodemographic and Personal Medical History for EDS

n = 121		Mean (SD) / n	Median (P25-P75) / (%)
Age		40.2 (10.7)	41.5 (33.5 – 46.5)
Age at symptom onset		14 (11.0)	12 (6 – 17)
Age at diagnosis EDS		37 (11.0)	40 (30 – 43)
BMI (kg/m2)		23.0 (4.5)	21.9 (20.0 – 25.2)
BMI status			
Underweight (< 18.5)		13	10.7
Normal weight (18.5 – 25.0)		76	62.8
Overweight (25.0 – 30.0)		26	21.5
Obesity (> 30)		6	5.0
† Marital status			
With partner		76	62.8
Without partner		45	37.2
Number of children		0.93 (1.0)	1.0 (0 – 2)
Educational Status			
Undergraduate		31	25.6
Graduate		59	48.8
Postgraduate		19	15.7
Others		12	9.9
‡ Work status			
Employed		17	13.5
Unemployed		109	86.5
Personal medical history			
EDS			
Hypermobile		73	60.3
Classic		12	9.9
Vascular		2	1.7
Other		11	9.1
Unknown		23	19.0
Number of food allergies		1.2 (1.6)	1 (0 – 2)
Food allergies	No	59	48.8
	Yes	62	51.2
Dysautonomia	No	102	84.3
	Yes	19	15.7
Fibromyalgia	No	99	81.8
	Yes	22	18.2
Obesity	No	95	78.5
	Yes	26	21.5
Anorexia	No	102	84.3
	Yes	19	15.7
Bulimia	No	106	87.6
	Yes	15	12.4

*EDS* Ehlers-Danlos Syndrome, *n * sample, *%* percentage, *SD* standard deviation, *P25* percentile 25, *P75* percentile 75, *BMI * Body Massa Index

^†^ With partner Married/living, without partner Single/separated/divorced/widowed

^‡^ Employed Working, Unemployed studying/unemployed/retired/ disability/illness/housewife

Table 2 shows the clinical variables of the participants. The SQCH score was m = 5.6 SD (1.2), and the uncontrolled eating factor of the TFEQ was the highest, m = 19.6 SD (6.2). 36.4% of the participants were at high risk of eating disorder (SCOFF ≥ 2). The total score of gastrointestinal problems assessed with the PAGI-SYM was m = 41.2 SD (21.2), with highest scores in the subscale of heartburn symptoms: m = 13.2 SD (9.0).

**Table 2:** Clinical variables

n = 121	Mean (SD)/n	Median (P25 – P75)/%	α
SQCH	5.6 (1.2)	6 (5 – 7)	0.40
TFEQ-Restricted eating	15.4 (5.5)	15 (11 – 20)	0.85
Body Satisfaction	5.0 (2.0)	5.0 (4 – 6)	0.66
SCOFF	1.3 (1.3)	1 (0 – 2)	0.57
Risk of ED (SCOFF ≥ 2)	44	36.4	
OHIP-49	18.3 (6.8)	18 (14 – 23)	0.89
PAGI-SYM	41.2 (21.2)	41 (23 – 57)	0.94
Heartburn	13.2 (9.0)	13 (5 – 20)	0.91
Postprandial fullness	8.1 (4.7)	8 (4 -12)	0.80
Nausea/vomiting	4.1 (3.9)	3 (0 -7)	0.85
Bloating	6.3 (3.1)	7 (5 – 8)	0.91
Upper abdominal pain	4.6 (3.0)	5 (2 – 7)	0.91
Lower abdominal pain	4.7 (2.8)	5 (2 – 7)	0.95
SSAS	36.7 (6.2)	37 (33 – 41)	0.71
Painful eating	2.1 (1.1)	2 (1 – 3)	
Fearful eating	1.6 (1.2)	2 (1 – 2)	
Avoidant eating	1.4 (1.1)	1 (0 – 2)	

n = sample; *%* percentage, *SD* standard deviation, *P25* percentile 25, *P75* percentile 75, *α* Cronbach’s alpha, *SQCH* Self-reported Screening Questionnaire for Joint Hypermobility Syndromes, *TFEQ* Three-Factor Eating Questionnaire, *ED* Eating disorder, *OHIP-49* Oral Health Impact Profile, *PAGI-SYM* Patient Assessment of Upper Gastrointestinal Disorders-Symptom Severity Index, *SASS* Somato-Sensory Amplification Scale

Table 3 shows the correlations between the predictors. High and positive correlations were observed between gastrointestinal problems and somatosensory amplification, oral pain, painful eating, fearful eating, and avoidant eating. The variance inflation factor (VIF) was < 2.5, indicating no collinearity.

**Table 3:** Correlations between variables

	1	2	3	4	5	6	7	8	9
1. Number of allergies									
2. BMI	− 0.142								
3. PAGI-SYM	0.154	0.034							
4. SSAS	− 0.077	0.095	0.284*						
5. Oral pain	0.044	0.054	0.512*	0.270*					
6. Painful eating	0.208*	− 0.031	0.527*	0.157	0.402*				
7. Fearful eating	0.309*	− 0.088	0.463*	0.180*	0.351*	0.677*			
8. Avoidant eating	0.277*	– 0.125	0.381*	0.169	0.298*	0.570*	0.846*		
9. Body satisfaction	0.164	− 0.370*	− 0.130	− 0.154	− 0.237*	− 0.001	0.051	0.060	
10. TFEQ- Restricted eating	− 0.096	0.287*	0.028	0.196*	0.057	0.050	0.094	0.134	− 0.544*

Spearman correlations; * p ≤ 0.05. Variance Inflation Factor (VIF) < 2.5

*BMI* Body mass index, Gastrointestinal problems = PAGI-SYM Patient Assessment of Gastrointestinal Symptom Severity Index; Somatosensory amplification = SASS Somato-Sensory Amplification Scale score; Oral pain = physical pain dimension of the Oral Health Impact Profile (OHIP-49); TFEQ-Restricted eating = restricted eating factor of the Three-Factor Eating Questionnaire

Table 4 shows the structural model, with both direct standardized and unstandardized effects. The initial model, which was adapted from a previous theoretical approach [15], did not reach an adequate criterion fit. Using MIs, we added two pathways, one from gastrointestinal problems to oral problems, and another from allergies to fearful eating. The final model showed excellent goodness of fit indices; Chi Squared = 26.26 df (28), p = 0.5589; Root Mean Squared Error of Approximation (RMSEA) = 0.000 (90CI% 0.000–0.065); Comparative Fit Index (CFI) = 1.000; Tucker-Lewis Index (TLI) = 1.000; Standardized Root Mean Squared Residual (SRMR) = 0.062; Coefficient of Determination (CD) = 0.406.

**Table 4:** ED Structure Model. Standardized and unstandardized effects

Direct effects		Standardized effects			Unstandardized effects		
Outcome	Predictor	Coef	CI95%	P	Coef	CI95%	p
Allergies	Somatosensory amplification	0.04	− 0.14 – 0.21	0.690	0.01	− 0.04 – 0.06	0.691
	cons	0.52	− 0.55 – 1.60	0.339	0.84	− 0.88 – 2.57	0.337
Gastrointestinal Problems	Allergies	0.18	0.02 – 0.34	0.032	2.36	0.17 – 4.56	0.035
	Somatosensory amplification	0.30	0.14 – 0.45	< 0.001	1.01	0.44 – 1.58	< 0.001
	cons	0.05	− 0.95 – 1.06	0.919	1.11	− 20.19 – 22.40	0.919
Oral pain	Gastrointestinal problems	0.57	0.45 – 0.69	< 0.001	0.18	0.13 – 0.23	< 0.001
	cons	1.57	1.10 – 2.03	< 0.001	10.69	8.51 – 12.87	< 0.001
Painful eating	Allergies	0.08	− 0.06 – 0.23	0.294	0.05	− 0.05 – 0.15	0.291
	Gastrointestinal problems	0.42	0.24 – 0.59	< 0.001	0.02	0.01 – 0.03	< 0.001
	Oral pain	0.19	0.02 – 0.37	0.033	0.03	0.00 – 0.06	0.034
	Somatosensory amplification	0.02	− 0.13 – 0.18	0.764	0.00	− 0.02 – 0.03	0.764
	cons	0.42	− 0.49 – 1.34	0.364	0.45	− 0.50 – 1.40	0.355
Fearful eating	Allergies	0.13	0.00 – 0.23	0.047	0.10	0.00 – 0.20	0.043
	Painful eating	0.65	0.55 – 0.76	< 0.001	0.76	0.61 – 0.91	< 0.001
	cons	− 0.07	− 0.36 – 0.20	0.582	− 0.09	− 0.44 – 0.26	0.590
Avoidant eating	Fearful eating	0.85	0.80 – 0.90	< 0.001	0.78	0.70 – 0.87	< 0.001
	cons	1.57	1.10 – 2.03	0.221	0.11	− 0.06 – 0.28	0.201
Restricted eating	Avoidant eating	0.17	0.02 – 0.32	0.023	0.82	0.11 – 1.54	0.024
	Body satisfaction	− 0.54	− 0.67—-0.41	< 0.001	− 1.45	− 1.85—-1.05	< 0.001
	cons	3.94	3.47 – 4.43	< 0.001	21.46	19.16 – 23.77	< 0.001
BMI	Avoidant eating	− 0.11	− 0.28 – 0.05	0.182	− 0.45	− 1.12 – 0.22	0.184
	Body satisfaction	− 0.30	− 0.49—-0.11	0.002	− 0.66	− 1.11—-0.23	0.003
	Restricted eating	0.08	− 0.11 – 0.28	0.402	0.07	− 0.09 – 0.23	0.403
	cons	5.78	4.70 – 6.86	< 0.001	25.88	21.78 – 29.99	< 0.001

CI95% = confidence interval 95%; p = p value; Gastrointestinal problems = PAGI-SYM Patient Assessment of Gastrointestinal Symptom Severity Index; Somatosensory amplification = SASS Somato-Sensory Amplification Scale score; Oral pain = physical pain dimension of the Oral Health Impact Profile (OHIP-49); Restricted eating = restricted eating factor of the TFEQ Three-Factor Eating Questionnaire; *BMI * Body mass index; cons = constant

Goodness-of-fit: Chi Squared = 26.26 df (28), p = 0.5589; Root mean squared error of approximation (RMSEA) = 0.000 (90CI% 0.000–0.065); Comparative fit index (CFI) = 1.000; Tucker-Lewis index (TLI) = 1.000; Standardized root mean squared residual (SRMR) = 0.062; Coefficient of determination (CD) = 0.406

**Fig. 1:**
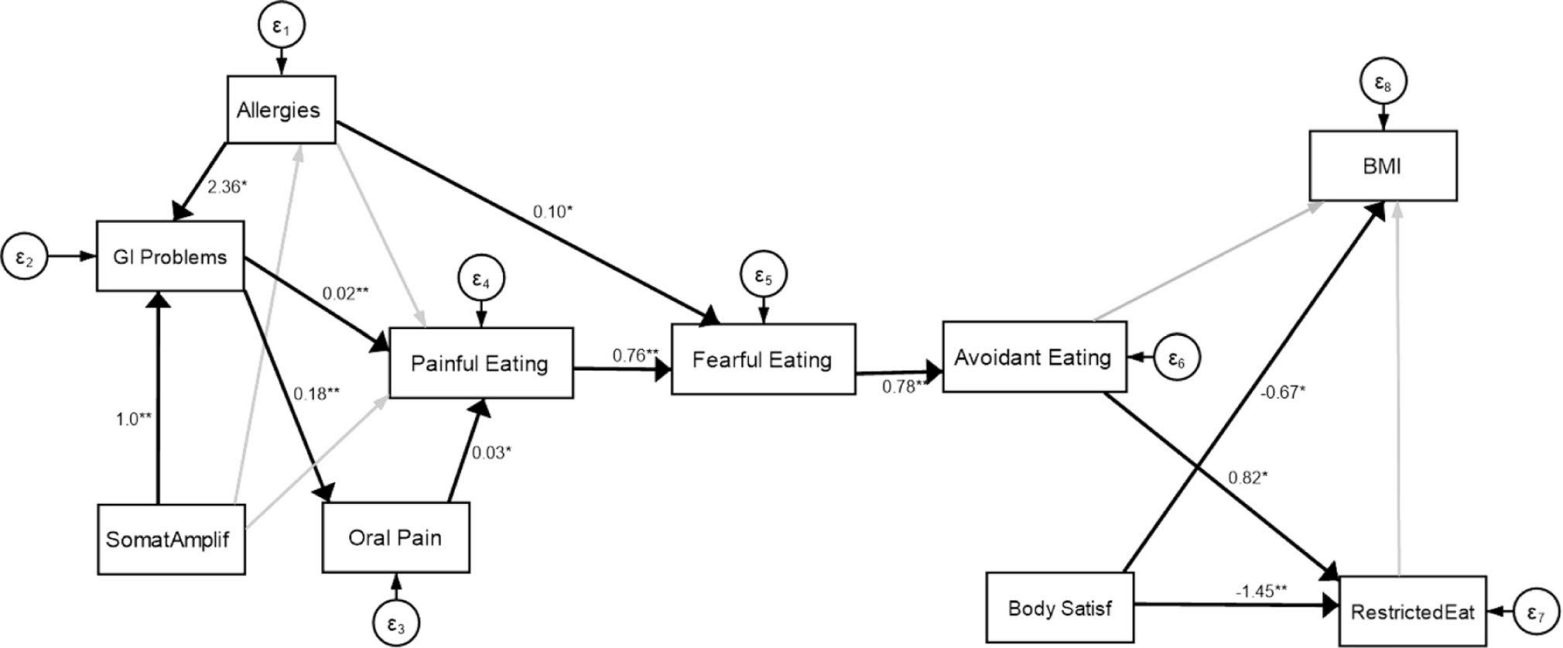
Structural model. Unstandardized coeficients. p < 0.05*, p < 0.001** *GI problems* Gastrointestinal problems = patient Assessment of Gastrointestinal Symptom Severity Index, *SomatAmplif * Somatosensory amplification , *SASS* Somato-Sensory Amplification Scale score, *Oral pain* oral pain dimension of the Oral Health Impact Profile (OHIP-49); RestrictedEat = Restricted eating = restricted eating factor of the TFEQ Three- Factor Eating Questionnaire, *Body Satisf* Body satisfaction; *BMI* Body mass index

Figure 1 with unstandardized coefficients shows different significant direct pathways. Firstly, between allergies and gastrointestinal problems (B = 2.36, p = 0.035) and allergies and fearful eating (B = 0.10, p = 0.043), as well as between gastrointestinal problems and painful eating (B = 0.02, p = 0.001) and oral pain (B = 0.18, p < 0.033). Second, somatosensory amplification showed a positive relationship with gastrointestinal problems (B = 1.01, p < 0.001). Third, there are positive associations between painful eating and fearful eating (B = 0.76, p < 0.001), as well as between fearful eating and avoidant eating (B = 0.78, p < 0.001). Finally, avoidant eating showed a positive relationship with restricted eating (B = 0.82, p = 0.024), whereas the relation between body satisfaction and restricted eating was negative (B = − 1.42, p < 0.001). Body satisfaction was also negatively associated with BMI (B = − 0.67, p = 0.002).

## Discussion

The aims of the present work were to explore a) some physical and psychological factors common in EDS (especially in hEDS) such as GI disturbances, food allergies, oral pain and somatosensory amplification, and b) their relationships with eating-related outcomes. The latter operationalized as painful eating, fearful eating, eating avoidance, food restriction, and BMI, allowing to test the exploratory hypothesis that altered eating behaviors in EDS may be secondary to physical and psychological features commonly associated with this condition [15].

First, it did not as a surprise the finding that a high percentage of participants present negative eating-related outcomes. For example, a high risk of ED and an abnormal BMI were observed in a third of participants. In addition, half of the sample declared suffered from food allergies. These results confirm previous reports from studies in samples from France, United States and United Kingdom [9, 19, 46]. Concerning the connections between the variables explored, we observed a link between somatosensory amplification, food allergies and GI problems. Indeed, although food allergies may affect many organs, several manifestations correspond to GI symptoms (e.g. nausea, diarrhoea, colic). On the other hand, somatosensory amplification which is the tendency to experience unpleasant bodily sensations as abnormally intense, noxious and disturbing [47], has been described as playing a role in the generation and perception of functional GI disturbances such as dyspepsia [48].

Moreover, we observed that GI problems predict oral pain, which is consistent with literature since it is well known that many GI disorders of varied aetiologies can lead to oral lesions with consequent pain [49]. These physical and psychological hEDS comorbidities, appear significantly linked to negative and dysfunctional sensations (pain), emotions (fear) and behaviours (avoidance and restriction) in the context of eating. Thus, in line with our hypothesis, this result can be presented as a parallelism of the fear-avoidance model of chronic pain [50], which is considered a “leading paradigm to understating disability associated with musculoskeletal conditions” [51]. This model formulates that pain-related fear trigger coping mechanisms that lead avoidance of movement and activity which may be perceived as useful in the short term, but in the long term perpetuates pain, negative mood and disability. Therefore, fear-avoidance model provides a useful framework to understand eating altered behaviours and its chronicity in some patients with hEDS. This purpose is supported by the results obtained by prior research [19], that confirm that altered diet and irregular eating patterns are frequent in patients with hEDS and hypermobility spectrum disorders, and that these problems are associated with GI problems such as dyspepsia and reflux, and ARFID. These authors also observed that fear of negative consequences from eating was strongly associated with restrictive behaviours such as skip meals and nutrition support.

Finally, no direct effect was found of avoidant or restricted eating on BMI; however an effect of body satisfaction on BMI was observed, which is consistent with results of a meta-analysis that conclude a negative association between body appreciation and BMI in females [52]. These results should be interpreted with caution, as self-reported BMI introduces potential measurement bias, as participants may underreport weight or overreport height in social desirability, possibly leading to systematic underestimation of BMI.

## Limitations and strengths

Regarding the limitations, it should be noted that due to the cross-sectional design of the present study, we could only assess associations and direct effects among the variables of interest, but not mediation effects of painful, fearful and avoidant eating. Future research should focus on longitudinal studies that allow for establishing a temporal sequence and explore these mediation effects in depth. In addition, the sample size is relatively small, and participants were recruited based on a self-reported diagnosis of EDS. However, an instrument was used to assess JH syndrome (SQCH) [25] which before the EDS classification published in 2017 [2] was considered a clinical picture that overlap with hEDS [53]. The results of this measure showed a very high score (5.45 out of 7 on average) confirming the important symptomatology experienced by participants. Lastly, while self-report data are generally acceptable for capturing subjective symptom experiences, it is less reliable than clinician-made or clinician-confirmed diagnoses of complex medical conditions and disorders (e.g., fibromyalgia, dysautonomia, anorexia nervosa, bulimia nervosa…), including objective anthropometric measurements such as height and weight used to calculate BMI. Future research shall consider the potential benefits of methodologically stronger designs that incorporate clinically verified diagnoses to allow more precise data collection and analytical subgroup comparisons.

As strengths, and to the best of our knowledge, this is the first model supporting empirically a plausibly pathway to understand altered eating behaviors in people with EDS, especially hEDS. This path may help understand the difficulties of a subgroup of patients. However, other trajectories might be identified. For instance, Gibson and Mehler (2024) [54] hypothesized that abnormal connective tissue develops because of ED manifestations such as starvation and weight loss. Thus, further studies are needed to confirm our results as other possibles mechanisms underlying the co-occurrence of connective tissue disorders and altered eating behaviors.

Some clinical considerations are worth mentioning. The painful symptomatology of hEDS goes beyond the musculoskeletal system, and extends to visceral, oral, and other locations. In that context, activities of daily life including feeding may be negatively impacted. In addition, there is evidence that individuals with different ED present eating related fears and avoidance behaviours [55]. Thus, the exploration of fears related to feeding and altered eating patterns is necessary in hEDS to prevent nutritional problems and ED. In this regard, clinicians should be aware of the overlap between hEDS and ED, particularly in the case of the most severe, functionally impairing and life-threatening EDs such as anorexia nervosa [54], and be aware of potentially atypical presentations or atypical forms of common ED symptoms (e.g., relatively uncommon gastrointestinal symptoms, dysautonomia, atypical or extreme expressions of fatigue and/or cognitive problems…) in order to ensure accurate diagnosis and clinical management in all patients.

## Data Availability

The data that support the findings of this study are available from the corresponding author upon reasonable request.
